# Genito-Urinary Surgery

**Published:** 1904-09-24

**Authors:** 


### GENITO-URINARY SURGERY.

(*Concluded from page* 416.)

**Perineal Litholopaxy.**—This procedure has been discussed by A. Hooton.[16] A curved staff with a median groove is passed into the bladder and a small incision made with a tenotome or small double-edged scalpel, about an inch in front of the anus in children, and this is deepened until the groove of the staff is entered. A director is next passed into the bladder through the wound, the staff withdrawn and dilators such as Hegar's introduced up to the required size. The bladder is then injected and the lithotrite used. Recovery is very rapid after the operation and often only a few drops of urine escape from the wound. The operation is recommended for stones requiring a lithotrite of a size not easily passable by the natural route, when stricture of the urethra is present or the urethra is narrow, and when a small lithotrite is not obtainable in a case requiring such for ordinary litholopaxy.

**Urinary Tuberculosis.** — The treatment of this disease was recently discussed in the Medical Society of London.[17] E. Hurry Fenwick recommended the use of Koch's new tuberculin for early cases in which the mucous membrane of the bladder was alone affected by the disease. He did not claim that a cure resulted from its use, but that it gave real relief from pain. It should not be given unless tubercle bacilli were present in the urine and its effects were to be carefully watched, for it might light up trouble in one or other kidney latently affected with tuberculosis. If the cystoscope showed one ureter "condensed and choked" by peri-ureteritis he advocated removing the corresponding kidney, and gave a list of 25 nephrectomies with one death. The heavy death-rate of nephrectomy for tuberculosis was due to the operation not being performed early enough. In the rare cases in which there was a single tubercular ulcer in the bladder he advised suprapubic excision. In the male the testis or testes, vasa deferentia and seminal vesicles must be sacrificed if diseased; but if the deposits of tubercle were many and widespread only hygienic, dietetic, and therapeutic measures were available. A. E. Wright remarked that the new tuberculin consisted of the protoplasm of the tubercle bacilli, and was a "vaccine" against tuberculosis. He had treated several cases of the severer forms of tuberculosis with repeated injections of tuberculin, and had thus successfully built up immunity to the disease. F. Swinford Edwards had found the tuberculin give much relief and in some cases effect a cure in tuberculosis of the urinary passages, but he had never knowingly tried it for tubercular disease of the kidney.

**Anatomy of the Normal and Enlarged Prostate.**—This subject was discussed at a meeting of the Royal Medical and Chirurgical Society on March 22nd, 1904. J. W. T. Walker described first the anatomy of the normal gland. The sheath of the pelvic fascia completely surrounded the prostate, except at the base and apex. The base was in direct relation with the circular bladder muscle. The apex was surrounded by a circular layer of striped muscle, continuous below with the constrictor urethra, and this layer of muscle passed up between the sheath and the capsule anteriorly. The veins of the prostatic plexus lay between the layers of the sheath and were separated from the capsule by a layer of the sheath, and in front also by the muscular layer mentioned. The prostatic sheath was much thicker and denser when the gland was enlarged than when it was of normal size. The vesiculæ seminales were pushed backwards away from the bladder when the prostate enlarged, and the urethra in the extra-vesical part of the gland sank backwards to the posterior surfaces of the lobes, probably owing to the drag of the ejaculatory ducts. There was no formation of a capsule of compressed gland substance. The cavity left after supra-pubic prostatectomy was formed posteriorly by the seminal vesicles enclosed in recto-vesical fascia, and lower down by a continuation of that fascia; at the sides the cavity was formed by the pelvic fascia, with the levator ani muscles outside; and in front by the layers of fascia containing the vertical part of the prostatic plexus. An examination of 73 specimens of the prostate removed by Mr. P. J. Freyer, showed that the line of cleavage passed between the prostatic capsule and the prostatic sheath of recto-vesical fascia. The fact that sometimes a shred of prostatic tissue was left behind did not invalidate this conclusion. R. Harrison said that he had found the operation in some cases to be a complete prostatectomy, in others the gland had been shelled out, leaving a distinct capsule of prostatic tissue behind.

**Prostatectomy.**—H. W. Allingham[19] records three cases of complete prostatectomy. The hæmorrhage in each case was trifling and there was very little shock. One patient died from bronchitis; the other two made excellent recoveries, though one of the latter was 80 years of age. James E. Moore (Minneapolis)[20] writes that prostatectomy is altogether too grave an operation to be resorted to as a routine treatment for every enlarged prostate. The most frequent cause of death after the operation is uræmia, and the operation is contra-indicated if there is evidence beforehand of serious kidney disease. The next greatest danger is sepsis, and the patients, being old, have diminished resisting power against sepsis. The only satisfactory treatment for cystitis complicating enlarged prostate is drainage, and this is best brought about by perineal prostatectomy. It is not reasonable to expect that there will not be a traumatic stricture later in those cases in which the prostatic urethra is sacrificed. In the perineal operation the most difficult problem is how to avoid injury to the rectum. One must keep well within the capsule of the gland. It is certain that with the present technique (perineal) the seminal ducts are sacrificed, and a technique will have to be adopted which does not sacrifice them if we hope to establish early operation for prostatic enlargement. Dribbling or incontinence occasionally follows the operation, from injury to muscles or nerves at the neck of the bladder. Sometimes a communication is formed between the rectum and urethra, requiring a plastic operation for its closure. Epididymitis is a common sequel of perineal prostatectomy and is sometimes so troublesome that the epididymis has to be removed.

[16] Brit. Med. Jour., April 9, 1904, p. 833. [17] Abst. Lancet, April 2, 1904, p. 935. [18] Abst. Ibid., March 26, 1904, p. 869. [19] Ibid., May 7, 1904, p. 1279. [20] Annals of Surgery, March 1904, p. 386.

